# Chronic palmitoylethanolamide administration *via* slow-release subcutaneous pellets promotes neuroprotection and mitigates neuroinflammation in the Tg2576 mouse model of Alzheimer’s disease

**DOI:** 10.3389/fncel.2025.1571428

**Published:** 2025-04-17

**Authors:** Daniel Tortolani, Davide Decandia, Giacomo Giacovazzo, Lucia Scipioni, Anna Panuccio, Francesca Ciaramellano, Fabiola Eugelio, Federico Fanti, Emanuele Claudio Latagliata, Livia La Barbera, Debora Cutuli, Dario Compagnone, Marcello D’Amelio, Roberto Coccurello, Sergio Oddi, Laura Petrosini, Mauro Maccarrone

**Affiliations:** ^1^European Center for Brain Research, Fondazione Santa Lucia IRCCS, Rome, Italy; ^2^Department of Veterinary Medicine, University of Teramo, Teramo, Italy; ^3^Department of Psychology, University Sapienza of Rome, Rome, Italy; ^4^Department of Biotechnological and Applied Clinical Sciences, University of L’Aquila, L’Aquila, Italy; ^5^Department of Bioscience and Technology for Food, Agriculture and Environment, University of Teramo, Teramo, Italy; ^6^Department of Medicine and Surgery, Università Campus Bio-Medico di Roma, Rome, Italy; ^7^Institute for Complex Systems (ISC), National Council of Research (CNR), Rome, Italy

**Keywords:** Alzheimer’s disease, ultramicronized palmitoylethanolamide, oxidative stress, neuroinflammation, dendritic spine density, memory

## Abstract

Alzheimer’s disease (AD) is a progressive neurodegenerative disorder characterized by cognitive and non-cognitive decline associated with neuropathological hallmarks, including neuroinflammation. Palmitoylethanolamide (PEA), an endogenous lipid with anti-inflammatory and neuroprotective properties, has emerged as a promising therapeutic agent in managing AD. This study investigated the therapeutic effects of chronic (6-months) PEA administration *via* subcutaneous pellet in Tg2576 mice, a validated model of AD. The impact of PEA on amyloid precursor protein (APP) processing, astrocytic activation, microglial reactivity and neuroinflammation, nitrosative stress, dendritic spine density in hippocampal CA1 pyramidal neurons, and cognitive performance was assessed. Chronic PEA treatment of Tg2576 mice increased the expression of the α-secretase ADAM9 and reduced astrogliosis. Furthermore, PEA attenuated microglia reactivity, downregulated pro-inflammatory (CXCL13, MCP-1, GCSF) and upregulated anti-inflammatory (CXC3CL1 and IL-9) cytokine expression. Chronic PEA administration also decreased protein nitrosylation, downregulated calcineurin expression, restored dendritic spine density, and improved cognitive functions. Chronic PEA administration offers a promising therapeutic approach for AD by mitigating neuroinflammation, oxidative stress, and synaptic dysfunction, ultimately leading to cognitive function restoration.

## Introduction

Alzheimer’s disease (AD) is one of the most critical healthcare challenges of our times, with its prevalence expected to rise dramatically in the coming decades. Characterized by progressive cognitive and non-cognitive decline and neuronal degeneration, AD poses a significant burden on affected individuals, caregivers, and society.

In addition to the classical amyloidogenic and tau phosphorylation hypotheses, the investigation of AD pathogenesis now considers the impact of sustained neuroinflammation as a key driver of AD progression. Indeed, it is now believed that AD is an amyloid-initiated tauopathy with neuroinflammation serving as the link between amyloid deposition and neurodegeneration. This concept posits that chronic neuroinflammation plays a pivotal role in the pathogenesis and progression of AD (Minter et al., 2016; Leng and Edison, 2021; Thakur et al., 2023). Indeed, chronic activation of microglia and astrocytes contributes to the accumulation of amyloid plaques, the hallmark neuropathological feature of AD (Singh, 2022; Twarowski and Herbet, 2023). In particular, microglia are also critical players in the initiation of plaque formation. Specifically, microglial uptake and aggregation of apolipoprotein E (APOE), a glycoprotein involved in lipid transport and metabolism, can trigger Aβ amyloidosis within the endo-lysosomal system (Kaji et al., 2024). Furthermore, studies using colony-stimulating factor 1 receptor (CSF1R) inhibition to deplete microglia have demonstrated their essential role in plaque pathogenesis, revealing that plaque formation is largely dependent on the presence of these cells (Spangenberg et al., 2019). Activated immune cells in the brain also release a plethora of pro-inflammatory mediators, including cytokines, chemokines, and reactive oxygen species, which can damage neurons and impair synaptic functions (Minter et al., 2016; Leng and Edison, 2021; Twarowski and Herbet, 2023). Moreover, neuroinflammation can disrupt blood–brain barrier integrity, leading to increased infiltration of peripheral immune cells and influx of pro-inflammatory interleukins and other inflammatory proteins into the brain, thus further exacerbating central inflammation (Chen and Yu, 2023). Of note, genetic studies have identified variants in genes related to immune function as risk factors for AD (Prendecki et al., 2020).

Considering the strong link between neuroinflammation and AD, targeting neuroinflammatory pathways has emerged as a promising therapeutic strategy. In this context, it might be particularly relevant to design dietary interventions in mild-cognitive-impairment (MCI) and AD subjects through nutraceuticals, such as lipid molecules that may play a primary role to fight, or at least, delay chronic neuroinflammation and, thus, halt or slow down the conversion from MCI to AD.

For instance, palmitoylethanolamide (PEA) appears of interest due to its high efficacy/risk ratio and the lack of both tolerance induction and crosstalk with other conventional therapies used for counteracting mental decline (Beggiato et al., 2019; Bortoletto et al., 2022; Bossa et al., 2023). PEA is an endogenous bioactive lipid that is biochemically and functionally related to endocannabinoid signaling, because it: (i) has a low affinity for the cannabinoid receptors 1 and 2 (CB_1_ and CB_2_), and (ii) enhances the activity of the main endocannabinoid anandamide (arachidonoylethanolamide, AEA), through an “entourage effect” (Solorzano et al., 2009; Landolfo et al., 2022). Within the brain, PEA is produced “on demand” by neurons, microglia, and astrocytes, thus having a pro-homeostatic role in neurochemical and neuroimmune responses to various detrimental processes, including those associated with AD (Solorzano et al., 2009; Landolfo et al., 2022).

Against this background, the present study investigates the effects of long-term administration of ultra-micronized PEA (UM-PEA) in a validated mouse model of AD (i.e., Tg2576 mice). These transgenic mice overexpress a mutated form of the human amyloid precursor protein (APP) gene with the Swedish mutation (APPswe KM670/671NL), leading to the overproduction of β-amyloid (Aβ) peptides and their accumulation in amyloid plaques, a typical pathological hallmark of AD (Hsiao, 1998). Consequently, Tg2576 mice develop a range of AD-like behavioral and cognitive deficits when they reach a fully symptomatic phase. These deficits encompass memory impairment, spatial learning difficulties, and disrupted social interactions, closely mirroring the cognitive decline observed in AD patients (Hsiao, 1998; Scipioni et al., 2023).

## Materials and methods

### Animals

All experimental procedures were performed in strict compliance with the ARRIVE guidelines and were carried out according to the European Guidelines for Animal Research (2010/63/EU, Protocol: Maccarrone 421-2019-PR) and following the regulations set forth by the Italian Ministry of Health (L.D.26/2014). All the procedures also received approval from both the internal animal welfare office and the Department of Public Health and Veterinary. Transgenic mice (Tg2576) and their wild type (WT) counterparts were housed in the conventional animal facility of the IRCCS Santa Lucia Foundation (Rome, Italy). Food and water were made freely available to the mice, always ensuring ad *libitum* access. Mice were housed with a 12 h light/dark cycle. Tg2576 mice, along with WT mice, were group-housed (3–4 mice/cage) under controlled temperature (22–23°C) and humidity (60 ± 5%) conditions.

### Transgenic AD-like mouse model

Male transgenic Tg2576 mice expressing the mutated APP bearing the Swedish K670N/M671L mutation were used in the present study. These mice exhibit progressive cognitive decline, amyloid plaque aggregation in the brain, neuroinflammation, and synaptic loss (Hsiao, 1998). Tg2576 mice are heterozygous for the APP-K670N/M671L transgene and were generated by crossing hemizygous males (Tg2576-F0) with C57BL/6J/SJL-F0 hybrid females, obtained by crossing SJL males with wild type (WT) C57BL/6J females. Genotyping was performed between 20 and 25 days of age via polymerase chain reaction (PCR) analysis of tail biopsies to confirm the presence of the human mutant APP DNA sequence. Tails were collected from the mice to extract genomic DNA using standard techniques. Subsequently, 10 ng of DNA per sample was amplified by PCR. Genotyping was carried out using the following primers: (FW 5′-CTG ACC ACT CGA CCA GGT TCT GGG T-3′, REV 5′-GTG GAT AAC CCC TCC CCC AGC CTA GAC CA-3′; by Sigma Aldrich) (Maccarrone et al., 2018).

### Palmitoylethanolamide chronic delivery

Specifically, we utilized 90-day release drug pellets loaded with UM-PEA. UM-PEA and placebo were administered to both Tg2576 and WT mice *via* subcutaneously implanted micro-pellets (Innovative Research of America, IRA, Sarasota, Florida 34236, USA). These pellets contained the bioactive PEA integrated within a matrix consisting of cholesterol, cellulose, lactose, phosphates, and stearates. The placebo pellets shared an identical composition except for the active compound. Following the producer’s specifications, during the inclusion of UM-PEA in the drug pellet, the UM-PEA is distributed in the pellet matrix to keep its micrometric size and not alter its genuine crystalline form. Each drug pellet was customized to deliver 80 mg UM-PEA in 90 days, corresponding to approximately 0.88 mg/UM-PEA per day (i.e., 30 mg/kg). Both subcutaneous administration and dosage were selected based on previous investigations (Grillo et al., 2013; Guarnieri et al., 2014). Tg2576 and WT mice were subjected to chronic PEA subcutaneous delivery for 6 months, starting from 6 months of age up to 12 months of age. Animals were randomly assigned to four experimental groups, as follows: (1) WT-PEA; (2) WT-Placebo; (3) Tg2576-PEA; and (4) Tg2576-Placebo. Since drug pellets are designed to deliver continuously for 90 days, to obtain 180 days of chronic delivery, each mouse was implanted twice: the first pellet implanted at treatment day 0 delivered the drug until treatment day 90, while the second pellet implanted at treatment day 90 delivered the drug until treatment day 180. All animals whose brains were utilized for biochemical and histological analyses were previously subjected to behavioral testing using the Novel Object Recognition (NOR) test.

### Surgical procedures

Mice underwent intraperitoneal anesthesia using a solution containing 25 mg/mL ketamine, 2.5 mg/mL xylazine, and 14.25% ethanol in a sterile 0.9% NaCl solution, administered at a dose of 0.15 mL/20 g mouse. Following anesthesia, approximately 1 cm square of mid-dorsal skin was shaved, cleansed with ethanol, and coated with Betadine. Mice were then transferred to a procedural table that was cleaned with 70% ethanol solution and covered with a clean disposable towel. A sterile disposable blade was used to obtain a 4–5 mm incision through the skin. Sterile forceps were utilized to separate the skin and create an approximately 3 × 6 cm subcutaneous pocket. Drug pellets were placed onto the subcutaneous musculature, and the skin flaps were pulled together and stapled with 9 mm autoclips (KentScientific Torrington CN) (Guarnieri et al., 2014). Afterwards, the mice were relocated to a holding cage equipped with a 37°C heating pad and covered with a clean disposable towel. Mice were individually housed and transferred to a clean cage upon regaining consciousness. To ensure reliable and continuous performance, we implemented measures to avoid any contact between the pellets and organic solvents or external fluids during the implantation process. Mice were subjected to daily monitoring to verify the absence of visible edema or inflammation.

### Quantification of plasma and brain PEA levels by ultra-high-performance liquid chromatography-mass spectrometry (UPLC-MS/MS)

Brain and plasma levels of PEA and AEA were assessed using UPLC-MS/ MS as reported previously (Fanti et al., 2023). Each analytical standard was purchased in solution: PEA-d_4_ and AEA-d_4_ were at 1 mg mL^−1^ in EtOH. Briefly, total brain tissues were homogenized by sonication in ice with three 30-s cycles at 90% of power spaced out by 30-s pauses, to avoid overheating of the sample. The homogenate was centrifuged at 12,500 rpm for 12 min at 4°C; then 50 μL of supernatant was diluted with 50 μL of methanol (MeOH) acidified with 200 mM formic acid (HCOOH) and 100 μL of H_2_O. For plasma samples, 50 μL of plasma were collected and 125 μL of MeOH acidified with 200 mM HCOOH were added for protein precipitation. The resulting sample was centrifuged at 12,500 rpm for 12 min at 4°C. Following centrifugation, 175 μL of supernatant was removed and 75 μL of H_2_O was added. For sample clean-up, OMIX C18 pipette tips were used.

The μSPE procedure can be readily carried out through five main steps: (1) activation with 100 μL of MeOH; (2) conditioning with 100 μL of H_2_O:MeOH (50:50); (3) sample loading (200 μL of diluted brain homogenate); (4) washing out H_2_O:MeOH solution (90:10); and (5) elution with 50 μL of MeOH acidified with 10 mM HCOOH. The resulting eluate was then collected in HPLC vials with micro-volume inserts. UPLC-MS/MS analysis was performed by an Acquity UPLC H-Class system (Waters, Milford, Connecticut, USA) coupled with a Qtrap 4,500 mass spectrometer (Sciex, Toronto, ON, Canada) equipped with a Turbo V electrospray ionization source and operating in positive mode (ESI+). Separation of the analytes was performed by a Kinetex XB-C18 1.7 μm 100 × 2.1 mm column from Phenomenex (Torrance, CA, USA). Mobile phases were H_2_O and acetonitrile, both with a concentration of 0.01% *v*/*v* HCOOH. The concentrations of PEA and AEA were then expressed as ng per mg of tissue for brain samples and nM for plasma samples.

### Western blot

Frozen brain samples were lysed in ice-cold lysis buffer (10 mM EDTA, 50 mM Tris–HCl, pH 7.4, 150 mM sodium chloride, 1% Triton-X-100) in the presence of protease and phosphatase inhibitor mixture and then were sonicated in ice-cold at approx. 180 watts power in rounds of 30 s sonication/30 s rest for each cycle for 5 min. Protein amount was quantified by the Bio-Rad Protein assay (Bio-Rad Laboratories, Hemel Hempstead, UK). An equal amount of protein (50 μg) was loaded onto 10% sodium dodecyl sulfate–polyacrylamide gels and blotted onto 0.45 μm polyvinylidene fluoride sheets (Amersham Biosciences, Piscataway, NJ, USA). Membranes were blocked with 5% nonfat dried milk for 1 h, and then incubated overnight with the following primary antibodies: anti-β-Actin (mouse monoclonal, 1:1000; #A5441; RRID: AB_ 476744; Sigma Aldrich, Saint Louis, MO, USA); anti-GAPDH (mouse monoclonal, 1:1000; #sc-47724: RRID: AB_627678; Santa Cruz Technologies, Dallas, Texas, USA); anti-GFAP (mouse monoclonal, 1:1000; #SAB5201104; RRID: AB_ 2827276; Sigma Aldrich, Saint Louis, MO, USA); anti-Nitrotyrosine (mouse monoclonal, 1:500; #ab7048; RRID: AB_ 305725; Abcam, Cambridge, UK) anti-INOS (mouse monoclonal 1:1000; #MA5-17139; RRID: AB_2538610; Thermo Scientific, Waltham, MA, USA); anti-ADAM9 (rabbit polyclonal; 1:500; #2099; RRID: AB_2257809, Cell Signaling Technology, Danvers, Massachusetts, USA); anti-Calcineurin (rabbit polyclonal; 1:500; #07-1490; RRID: AB_1586914; Merck Millipore, Burlington, Massachusetts, USA). Detection was performed by using peroxidase-conjugated goat anti-mouse antibody (1.5000; #STAR137P; RRID: AB_2075637; Bio-Rad Laboratories, Hemel Hempstead, UK) and peroxidase-conjugated mouse anti-rabbit antibody (1:5000; #sc-2357; RRID:AB_628497; Santa Cruz Technologies, Dallas, Texas, USA) with the chemiluminescence detection system Lightwave Plus (GVS, Bologna, Italy). Chemiluminescence signals were detected by exposing blots to the C-DiGit blot scanner (LI-COR, Lincoln, NE, USA). Optical densities of protein bands were measured by using Image Studio Software 4.0.21 (LI-COR), and mean ratios between proteins and β-actin were reported. Membrane stripping was performed using Re-blot Plus strong antibody Stripping solution (Merck Millipore, Burlington, Massachusetts, USA) for 20 min.

### Immunofluorescence

Mice were sacrificed through dislocation, brains were removed, and the hemi-brains were post-fixed in paraformaldehyde at 4°C for 48 h and transferred in 30% sucrose in phosphate buffer (PB, 0.1 M, pH 7.4) at 4°C until sinking. Coronal sections (40 μm-thick) were cut with a cryostat, and slices were collected in PB-Sodium Azide 0.02% until used for experiments. For hippocampal Iba-1 staining, sections were pre-treated with hydrogen peroxide (Peroxide Block, 10%, ScyTek Laboratories, ACA-IFU, USA) for 10 min at RT, and, after two washes in PB, slices were incubated with primary antibody Iba-1 (1:800; #019-19741; RRID: AB_839504; FUJIFILM Wako, Japan) in PB containing 0.3% Triton X-100 overnight at 4°C. Then, the slices were washed three times in PB and incubated with secondary antibody Alexa Fluor-488 donkey anti-rabbit (1:200; A21206; RRID: AB_2535792; Thermo Fisher Scientific, USA) in PB containing 0.3% Triton X-100 for 2 h at RT. All sections were counterstained with DAPI (1:2000; #18860.01; Serva, Germany) for 5 min in PB and mounted using an anti-fade medium (Fluoromount, Sigma-Aldrich, MO, USA). The labeling specificity was confirmed by the omission of primary antibodies and the use of normal serum (negative controls).

### Stereological cell count

Every third slice of dorsal hippocampus was processed for immunofluorescence to unbiased estimate Iba-1^+^ microglia cells (Krashia et al., 2019). The boundaries of the hippocampus used for counting were defined with DAPI staining, and area distinction was performed according to published guidelines (Paxinos and Franklin, 2019). We applied an optical fractionator stereological design (mono-lateral count) using the Stereo Investigator System software version 2021 (MicroBrightField Europe EK). A stack of MAC 5000 controller modules (Ludl Electronic Products, Ltd) was interfaced with a Zeiss Microscope Axio Imager KMAT with a motorized stage and the Zeiss camera Axiocam 506 mono with Workstation High End PC. A 3D optical fractionator counting probe (x, y, z dimensions: 100 × 100 × 25 μm) was applied. The area of interest was outlined using the 5x objective and microglia cells were marked with a 40x objective.

Cell number was estimated according to the following formula:


$$N=SQ\times \frac{1}{ssf}\times \frac{1}{asf}\times \frac{1}{tsf}$$

where SQ represents the number of microglial cells counted in all optically sampled fields, ssf is the section sampling fraction, asf is the area sampling fraction, and tsf is the thickness sampling fraction.

### Morphological analysis

The morphology of Iba-1^+^ cells in the hippocampus was analyzed by Sholl analysis (Spoleti et al., 2022). Microglial cells were imaged with an optical microscope (DMLB; Leica, Wetzlar, Germany) equipped with a motorized stage and a camera connected to Neurolucida 7.5v software (MicroBright-Field, Vermont, USA) that allowed for quantitative 3D-analysis of the entire cell. Only non-overlapping cells that showed clear soma and branching contour were analyzed. Soma area and perimeter were measured and Sholl analysis was done. It includes counting the number of process intersections, nodes and endings, and process lengths at fixed distances from the soma in concentric circles originating from the soma (radii), spaced 10 μm apart. Analysis was done with a 100x-oil-objective. Overall, 9 representative cells per animal were selected randomly for analysis, and all data were subsequently averaged for each mouse.

### Cytokine protein array

RayBio^®^ C-Series mouse inflammation antibody array (AAM-INF-1-8; RayBiotech Life Inc., Peachtree Corners, GA, USA) was used to assess cytokines release in frozen brain samples. Samples were homogenized with 1 × Cell Lysis Buffer (kit supplied) in the presence of Protease Inhibitor Cocktail (Sigma Aldrich, Saint Louis, MO, USA) and centrifuged (14.000 × *g* for 30 min at 4°C). Then, the protein concentration was evaluated with a BCA Protein Assay Kit (23227; Thermo Scientific, Waltham, MA, USA) and each sample was diluted to a final concentration of 500 μg. The inflammation antibody array membranes were blocked (1 h, room temperature) and then incubated with 1 mL of sample overnight at 4°C. After incubation, the membranes were washed first with 1 × Wash Buffer I (kit supplied) and then with 1 × Wash Buffer II (kit supplied). Each membrane was covered with 1 mL of diluted Biotin-Conjugated Anti-Cytokine antibodies (kit supplied) and incubated for 90 min at room temperature. Afterwards, the membranes were washed as above and incubated for 2 h at room temperature with 1,000-fold diluted HRP-conjugated streptavidin (kit supplied).

Immunocomplexes were detected using a Detection Buffer (kit supplied) and visualized by using a C-DiGit blot scanner (LI-COR, Lincoln, NE, USA). C-DiGit images were analyzed by using Image Studio Software 4.0.21 (LI-COR). Table 1 shows the distribution of antibodies in the membrane of the cytokine protein array.

**Table 1:** Representative membrane with distribution of antibodies used for cytokine protein array.

Positive CTRL	Positive CTRL	Negative CTRL	Negative CTRL	Blank	CXCL13	CD30 ligand	CCL12	CCL24	Fas ligand	CX3CL1	GCSF
GM-CSF	INF-γ	IL-1 α	IL-1 β	IL-2	IL-3	IL-4	IL-6	IL-9	IL-10	IL-12 p40/p70	IL-12 p70
IL-13	IL-17A	CXCL11	CXCL1	leptin	LIX	XCL1	MCP-1	M-CSF	CXCL9	CCL3	MIP-1 γ
RANTES	CXCL12α	I-309	CCL25	TIMP-1	TIMP-2	TNF-α	TNF RI	TNF RII	Blank	Blank	Positive CTRL

### Golgi-Cox staining

Golgi-Cox staining of brain tissue was performed by using the FD Rapid GolgiStain TM Kit (FD NeuroTechnologies, Columbia, MD, USA), following the recommended manufacturer’s protocol. After mice were sacrificed through dislocation, the brains were promptly immersed in Golgi impregnation solution (A/B) in a plastic container. The impregnation solution was replaced after 24 h, and the brains were kept in the dark at room temperature for a total of 14 days. After this period, brain tissue was transferred to solution C of the FD Rapid GolgiStain TM Kit for 72 h. Solution C was replaced after 24 h. The brains were then frozen and sliced (slice thickness: 100 μm) using a cryostat (CM1860 UV, Leica, Wetzlar, Germany). Slices containing the hippocampus (AP: from −1.22 mm to −2.30 mm from bregma) (Vogt and Paxinos, 2014) were collected and mounted onto gelatin-coated microscope slides. Sections were initially rinsed in _dd_H2O for 4 min, then immersed in the staining solution (solution D/E) for 10 min. After another 4-min rinse in _dd_H2O, the sections were dehydrated sequentially in 50, 75, and 95% ethanol for 4 min each, followed by four immersions in 100% EtOH for 3 min each. Slides were then cleared with xylene (three times, 4 min each) before being coverslipped with Eukitt mounting medium. Subsequently, the slides were allowed to dry and stored in the dark at 4°C.

### Analysis of dendritic spine density

Quantification of dendritic spine density was performed utilizing an optical microscope (Axio Imager M2, Zeiss, Oberkochen, Germany) equipped with a motorized stage and a camera linked to Neurolucida 2020.1.2 software (MicroBright-Field, Vermont, USA). Dendrites were delineated alongside spines (with 100x oil-immersion objective lens), and subsequently, images were exported to Neurolucida Explorer 2019.2.1 (MicroBright-Field, Vermont, USA) for spine density analysis. The density of dendritic spines was assessed in the pyramidal neurons of hippocampal CA1 (Nobili et al., 2017). Forty dendritic segments (20–25 μm in length) were acquired for each experimental group, comprising 20 basal and 20 apical dendritic segments. Spine density was calculated by measuring the length of the dendritic segment and by counting the number of spines along it.

### Novel object recognition (NOR) test

NOR is a validated test designed to assess recognition memory in rodents by utilizing their inherent inclination to investigate unfamiliar stimuli (Cohen and Stackman, 2015). The experimental apparatus comprised a square chamber made of transparent Plexiglas (48.5 × 48.5 × 24.5 cm). The assessment protocol encompassed three 5-min phases: habituation, training, and test trial (de Bruin and Pouzet, 2006; Arsenault et al., 2011). During the habituation phase, mice were permitted to freely explore the chamber. After returning mice to their home cages for 3 min, in the training phase, mice were placed in the now-familiar chamber containing two identical objects [two triangles measuring 6 × 9.5 cm, made of white Plexiglas, fixed to a 4-mm thick square (6 × 6 cm) base]. Following a delay of 1 h, to assess recognition memory (test trial), mice were reintroduced into the chamber, which now contained one familiar object alongside a novel object [a white upturned L, 9.5 cm in height with a short side of 6 cm, fixed to a 4-mm thick square (6 × 6 cm) base]. Contact time was scored when the animal explored an object for at least 1 s. To counteract potential side bias, the location of the novel object was counterbalanced across trials such that it occurred equally often on the left or right side (among the different animals). Following each trial, the arena was cleaned using a 10% EtOH solution to minimize olfactory cues. Each trial was recorded with a video camera linked to a monitor and an image analyzer (EthoVision XT, Noldus, Netherlands). In the habituation phase, motor parameters as mean velocity (cm/s) and total distance (cm) were acquired. In the training phase, the duration of exploration of the two identical objects was evaluated. In the test trial, to quantify novelty preference, the duration of exploration for each object during the test session was evaluated, and a Novelty Preference Index was computed using the formula:


$$\mathrm{Novelty}\;\mathrm{Preference}\;\mathrm{Index}=\left(\frac{\mathrm{time}\;\mathrm{spent}\;\mathrm{exploring}\;\mathrm{the}\;\mathrm{novel}\;\mathrm{object}}{ime\;\mathrm{spent}\;\mathrm{exploring}\;\mathrm{both}\;\mathrm{object}}\right)\times 100$$

### Statistical analysis

Results are reported as mean ± standard deviation (S.D.) or standard error (SEM). Data were elaborated and analyzed statistically using the R Statistical Package (R version 4.4.0 release: 2024-04-24) within RStudio software (version 2024.04.0 + 735, release: 2024-04-29) with the following libraries: dplyr v1.1.4 (Wickham et al., 2020) for data manipulation and transformation; matrixStats v1.3.0 (Bengtsson et al., 2024) for operations on rows and columns of matrices during data analysis; ggplot2 v3.3.5 (Hadley, 2016) and ggpubr v0.6.0 (Kassambara, 2023) for creating and customized graphs suitable for visualizing data. Following the European recommendation 2010/63/EU regarding the protection of laboratory animals and the ARRIVE guidelines established by the NC3Rs (National Center for the Replacement, Refinement and Reduction of Animals in Research), an *a priori* power analysis was conducted using G*Power version 3.1.9.6 for sample size estimation based on data from a pilot study. Data were analyzed using a two-way ANOVA with genotype and treatment as the main factors. *Post hoc* tests were made using Tukey’s test for multiple comparisons. Differences were considered significant at the *p* < 0.05 level. The statistical methods used for each analysis are specified in the figure legends.

## Results

### Distinct impact of chronic PEA administration on endocannabinoid levels in WT and Tg2576 mice

**Figure 1:**
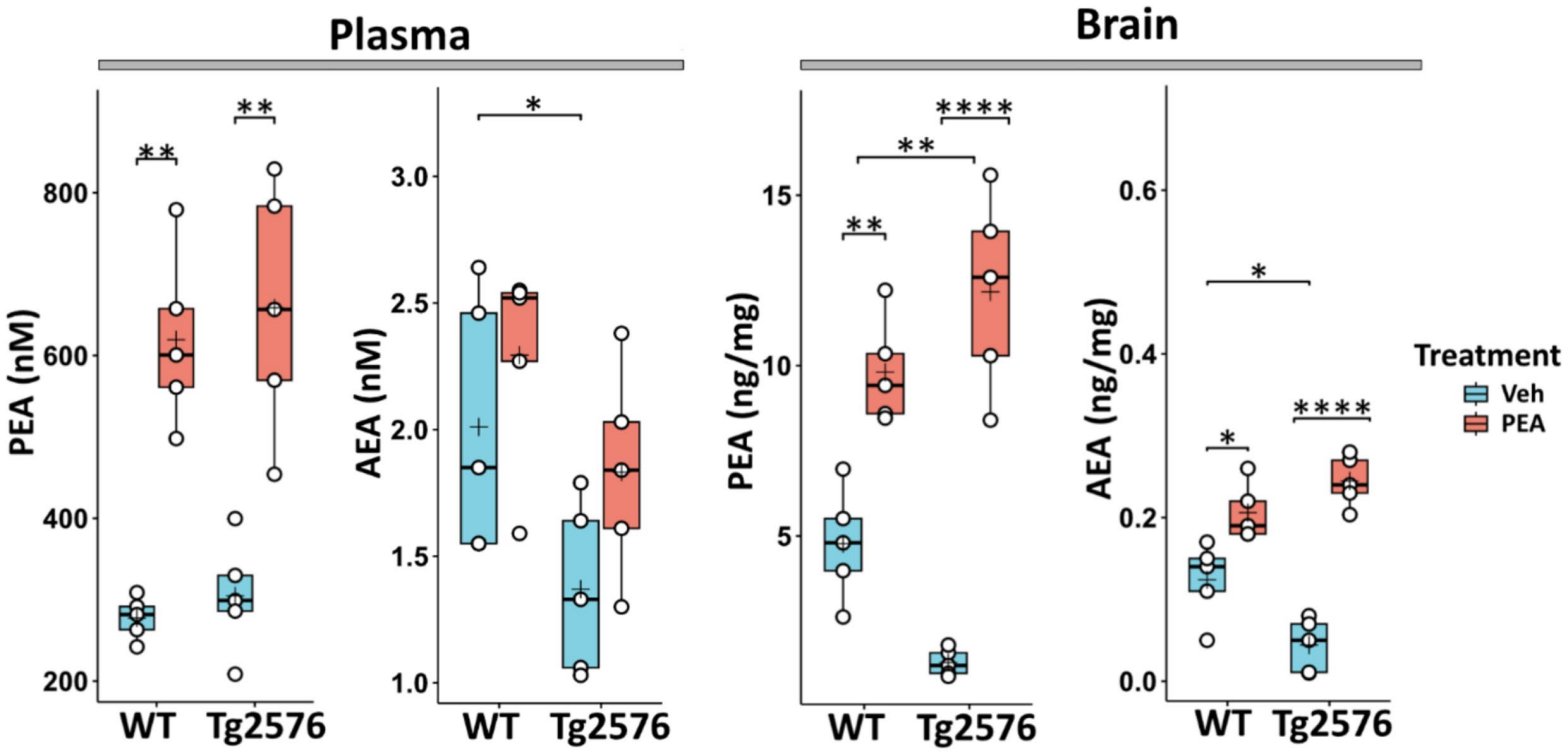
Plasma and brain levels of PEA and AEA in 12-month-old wild-type (WT) and Tg2576 mice after chronic PEA administration quantified by liquid chromatography-mass spectrometry. The horizontal line and + within the rectangle (boxplot) represent the median and the mean, respectively, of the values (white circles) which represent individual samples (*n* = 5 mice for each group). In this and in the following figures Veh indicates vehicle treatment and PEA indicates chronic PEA treatment. A two-way ANOVA was used to compare differences between treatment and genotype. Significance is shown as a *p* value, calculated using a *post hoc* Tukey’s test. *p < 0.05; **p < 0.01; ****p < 0.0001.

In a previous study (Piscitelli et al., 2019), the assessment of the plasma levels of PEA revealed that, compared to their non-transgenic littermates (WT mice), Tg2576 mice exhibited elevated PEA levels between 3 and 6 months of age. This time frame matches with the early symptomatic pre-plaque stages of APP-related amyloidosis. Subsequently, a decline in PEA levels was observed at 12 months of age, aligning with the symptomatic plaque-stage of the disease. Furthermore, at this age, Tg2576 mice displayed a reduction in PEA levels in both hippocampus and prefrontal cortex (Piscitelli et al., 2019). Thus, to increase PEA levels, we implanted a subcutaneous pellet that chronically released PEA from 6 months up to 12 months of age in both WT and Tg2576 mice. To assess the efficacy of chronic PEA administration, we measured plasma and total brain levels of PEA and its congener AEA in 12-month-old WT and Tg2576 mice (Figure 1). As expected, PEA administration significantly increased its circulating levels in both WT and Tg2576 mice (Figure 1, left), but it did not significantly impact on plasma AEA levels in both genotypes. Similarly, PEA administration resulted in a considerable increase in brain PEA concentrations in both WT and Tg2576 mice (Figure 1, right). Specifically, a ~ 2.2-fold increase in PEA levels was observed in WT mice upon PEA treatment compared to vehicle-treated controls (WT Veh: 4.77 ± 0.66 ng/mg; WT PEA: 9.80 ± 1.50 ng/mg; *p* = 0.0042), while Tg2576 mice demonstrated a more pronounced 9.4-fold elevation in PEA levels upon treatment (Tg Veh: 1.28 ± 0.39 ng/mg; Tg PEA: 12.10 ± 2.80 ng/mg; *p* < 0.0001). WT mice treated with PEA displayed a modest increase in AEA levels (WT Veh: 0.12 ± 0.04 ng/mg; WT PEA: 0.20 ± 0.03 ng/mg; *p* = 0.026). Tg2576 mice also showed a significant elevation in AEA levels upon PEA treatment (Tg Veh: 0.04 ± 0.03 ng/mg; Tg PEA: 0.24 ± 0.03 ng/mg; *p* < 0.0001). These findings indicate that PEA administration *via* subcutaneous pellets markedly affected the brain levels of both PEA and AEA, with a larger increase in Tg2576 mice.

### Chronic PEA administration affects the amyloidogenic pathway by inducing ADAM9 expression in Tg2576 mice

**Figure 2:**
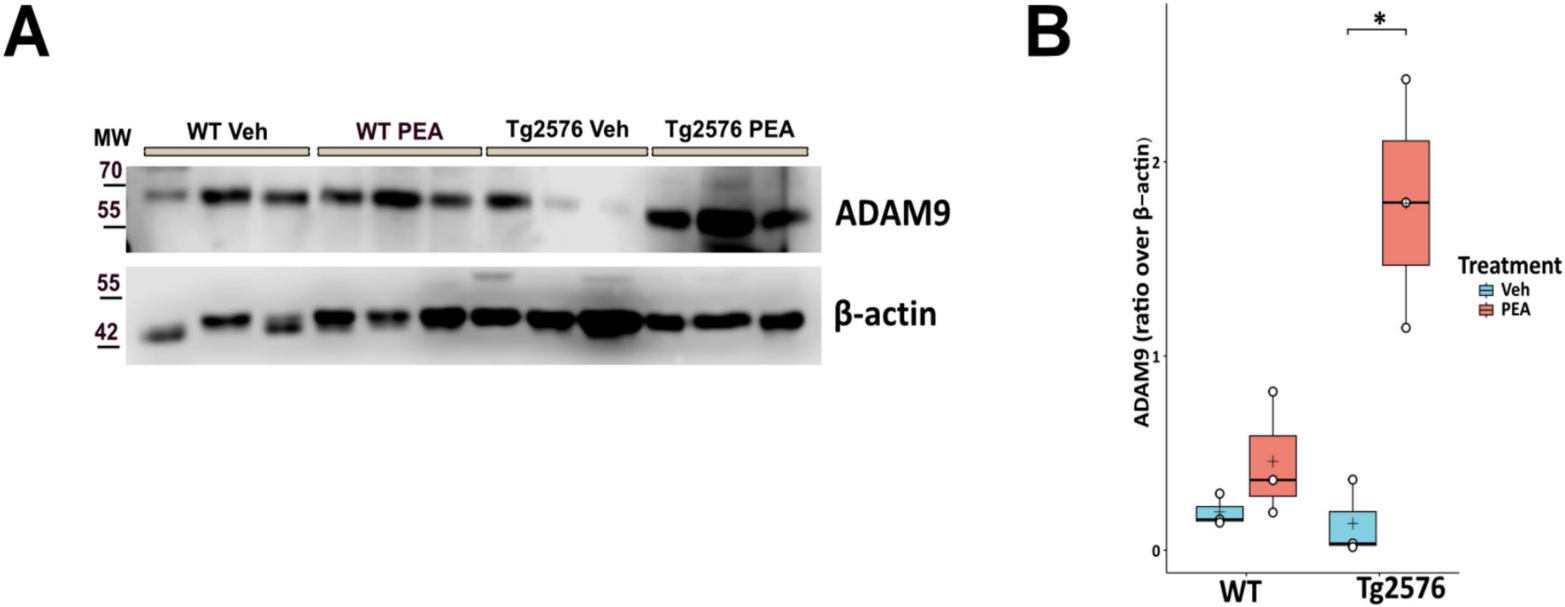
Expression of ADAM9 in the hippocampus of 12-month-old wild-type (WT) and Tg2576 mice upon chronic PEA administration. Western blot analysis of ADAM-9 in hippocampal extracts from WT and Tg2576 mice, β-actin was used as a loading control. **(A)** Representative immunoblots of ADAM9 in Tg2576 hippocampus, MW: molecular weight (kDa); **(B)** Horizontal line and + within the rectangle (boxplot) represent the median and the mean, respectively, of the densitometric analysis values (white circles) of the amounts of ADAM9 normalized to β-actin (*n* = 3 mice for each group). A two-way ANOVA was used to compare differences between treatment and genotype. Significance is shown as a *p* value, calculated using a post hoc Tukey’s test. *p = 0.013.

ADAM9 is a member of the ADAM (a disintegrin and metalloprotease) family, and plays a pivotal role in the processing of APP. This α-secretase is implicated in the non-amyloidogenic pathway of APP cleavage, thereby reducing the availability of substrates for β-secretase (BACE1) and γ-secretase (amyloidogenic pathway) (Asai et al., 2003; Chou et al., 2020). The ADAM9-mediated cleavage pathway mitigates amyloid-β (Aβ) peptide formation, a principal component of amyloid plaques (Asai et al., 2003). Here, we measured the hippocampal expression level of ADAM9 by Western blot analysis in both WT and Tg2576 mice, at the age of 12 months, after prolonged PEA treatment. Figure 2 shows that PEA treatment increases the expression of ADAM9 in the Tg2576 mice (Tg Veh: 0.14 ± 0.20; Tg PEA: 1.79 ± 0.64; *p* = 0.013; WT Veh: 0.20 ± 0.08; WT PEA: 0.46 ± 0.32) strongly suggesting that chronic PEA treatment exerts an anti-amyloidogenic action in Tg2576 mice.

### Chronic PEA administration markedly decreased astrogliosis in Tg2576 mice

**Figure 3:**
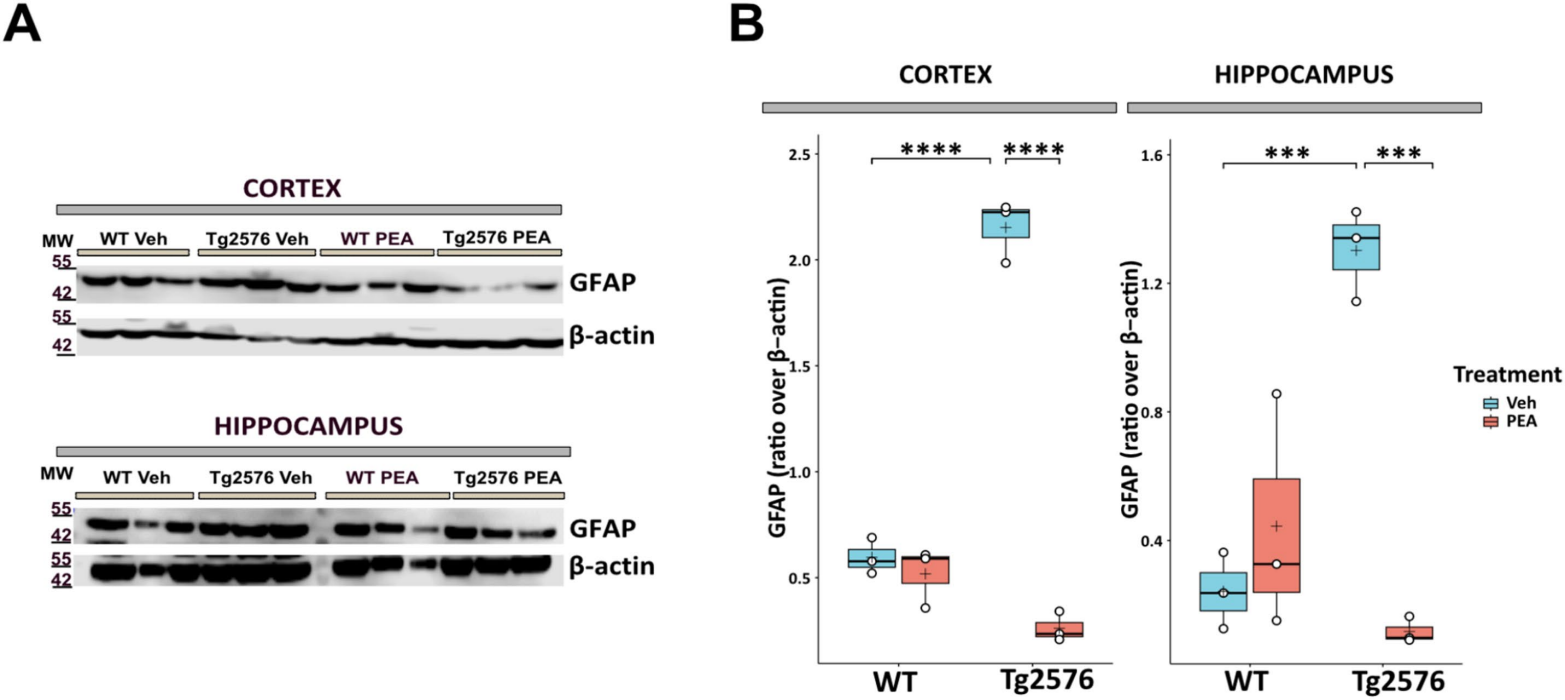
Expression of GFAP in the cortex and hippocampus of 12-month-old wild-type (WT) and Tg2576 mice upon chronic PEA administration. Western blot analysis of GFAP in cortical and hippocampal extracts from WT and Tg2576 mice, β-actin was used as a loading control. **(A)** Representative immunoblots of GFAP in the cortex (up) and hippocampus (down), MW: molecular weight (kDa); **(B)** Horizontal line and + within the rectangle (boxplot) represent the median and the mean, respectively, of the densitometric analysis values (white circles) of the amounts of GFAP normalized to β-actin (*n* = 3 mice for each group). A two-way ANOVA was used to compare differences between treatment and genotype. Significance is shown as a *p* value, calculated using a post hoc Tukey’s test. ***p < 0.001; ****p < 0.0001.

Astrogliosis is a significant pathological hallmark of AD, and the upregulation of glial fibrillary acidic protein (GFAP) – an indicator of its presence – is associated with the intensity of neuroinflammatory responses (Kumar et al., 2023). We measured the expression levels of GFAP within the hippocampus and cortex by Western blot analysis in both WT and Tg2576 mice, at the age of 12 months, after prolonged PEA or vehicle treatment. Figure 3 shows that GFAP protein levels were ~3.6-fold higher in the cortex and ~3.9-fold higher in the hippocampus of Tg2576 mice compared to WT mice (cortex: Tg Veh: 2.15 ± 0.14; WT Veh: 0.60 ± 0.08; *p* = 0.0001; hippocampus: Tg Veh: 1.30 ± 0.14; WT Veh: 0.24 ± 0.11; *p* = 0.0006). Notably, PEA treatment reduced the expression of GFAP levels in Tg2576 mice in both the examined brain regions (cortex: Tg Veh: 2.15 ± 0.14; Tg PEA: 0.26 ± 0.07; *p* = 0.00003; hippocampus: Tg Veh: 1.30 ± 0.14; Tg PEA: 0.12 ± 0.04; p = 0.0001).

### Chronic PEA administration modulated microglia morphology in Tg2576 mice

**Figure 4:**
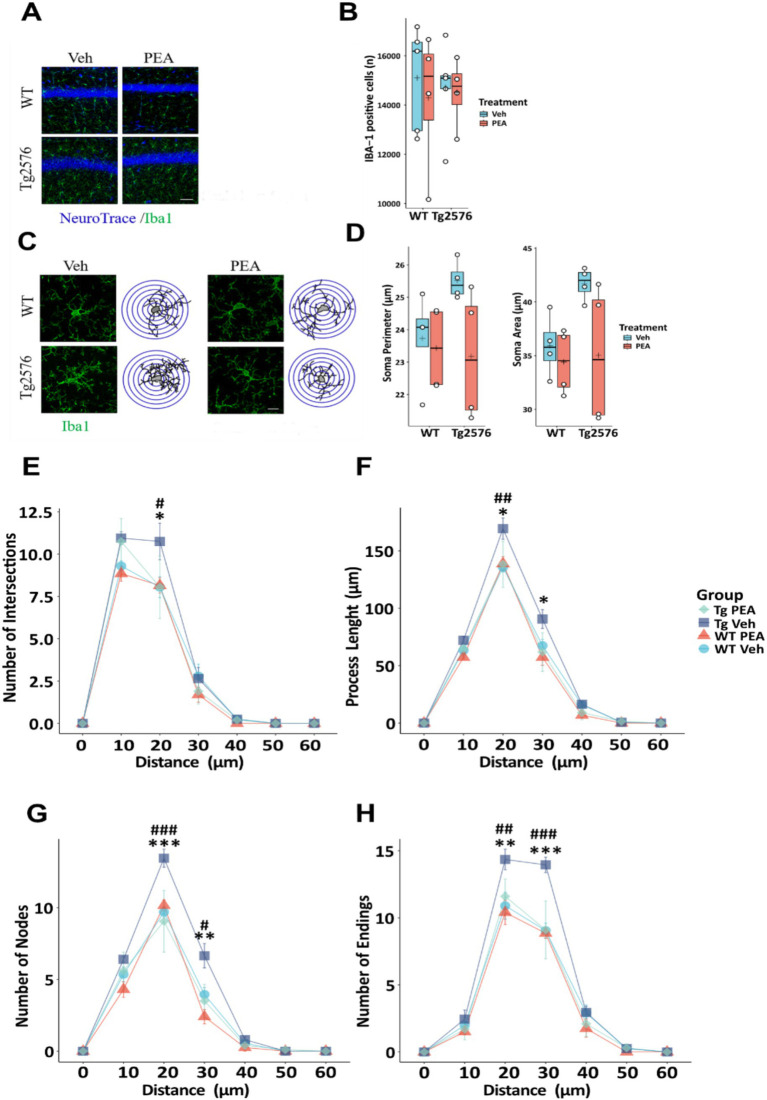
Stereological count of Iba-1 positive cells and microglia morphological evaluation of 12-month-old wild-type (WT) and Tg2576 mice upon chronic PEA administration evaluated by IBA-1 staining and Sholl analysis. **(A)** Representative ×20 objective images of Iba-1^+^ (green)/Neurotrace (blue) staining hippocampal regions used for analysis (scale bar: 50 μm). **(C)** Representative ×60 objective capture of cell morphology of an Iba-1^+^ cell (green) (scale bar: 10 μm), with the respective 3D-reconstruction by Sholl analysis. **(B,D)** Horizontal line and + within the rectangle (boxplot) represent the median and the mean, respectively, of the count **(B)** and soma parameters **(D)** of Iba-1^+^ cells (white circles) (*n* = 4 mice for each group). **(E–H)** Line plots with mean and SEM of morphological complexity of Iba-1 + cells depict **(E)** the number of intersections, **(F)** length of processes along radial distance from the soma, **(G)** number of nodes, and **(H)** number of endings. A two-way ANOVA was used to compare differences between treatment and genotype. Significance is shown as a p value, calculated using a post hoc Tukey’s test (Tg2576 Veh vs. Tg2576 PEA *p < 0.05; WT Veh vs. Tg2576 Veh #*p* < 0.05; Tg2576 Veh vs. Tg2576 PEA **p < 0.01; WT Veh vs. Tg2576 Veh ## *p* < 0.01; Tg2576 Veh vs. Tg2576 PEA ***p < 0.001; WT Veh vs. Tg2576 Veh ###*p* < 0.001).

In AD microglia cells become dysregulated, contributing to neuroinflammation and neuronal degeneration (Wang et al., 2016; Cai et al., 2022). To investigate microglial changes after chronic PEA administration, we first performed a count of Iba-1 positive (Iba-1^+^) cells using immunofluorescence staining (Figure 4A) and then quantified morphological changes of microglia by Sholl analysis. Stereological cell count revealed no significant differences in the number of Iba-1^+^ cells between WT and Tg2576 groups, under both vehicle and PEA treatment (Figure 4B). Microglial reactivity and severity of neuroinflammation are reflected by alterations in the morphology of the ramified processes (Figure 4C). Morphological assessment of microglia soma failed to reveal significant differences in somatic perimeter and area between WT and Tg2576 groups (Figure 4D), regardless of PEA treatment. To further investigate this aspect, we analyzed the process length, number of intersections, nodes, and endpoints in WT and Tg2576 microglia with chronic PEA or vehicle administration. As shown in Figures 4E–H, Tg2576 mice exhibited a significantly higher number of intersections, nodes, and endpoints, as well as longer processes, compared to WT mice. Notably, chronic PEA administration in Tg2576 mice resulted in a decreased number of intersections, nodes, and process endpoints, as well as shorter processes, bringing these parameters back to WT mice values.

### Effect of chronic PEA administration on the hippocampal inflammatory status of Tg2576 mice

**Figure 5:**
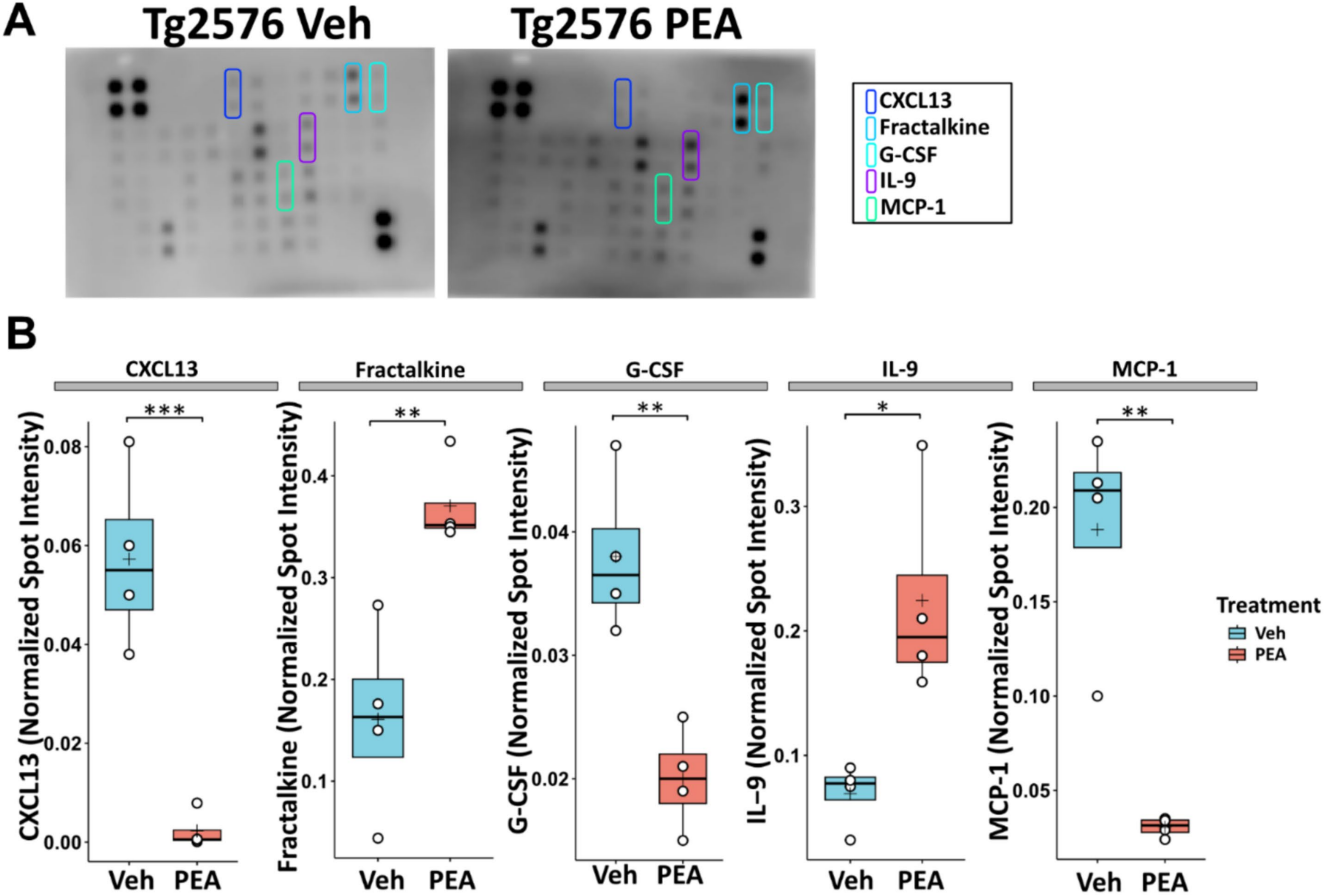
Inflammatory profile in the hippocampus of 12-month-old Tg2576 mice after chronic PEA administration. Protein microarray analysis in hippocampal extracts from Tg2576 mice treated with vehicle and PEA. **(A)** Representative array membranes that display significant changes in expression upon PEA treatment compared to the vehicle group. **(B)** The horizontal line and + within the rectangle (boxplot) represent the median and the mean, respectively, of the densitometric analysis of the normalized spot intensity values (white circles) (*n* = 4 array/mice for each group). Significance is shown as a *p* value, calculated using an unpaired t-test. *p = 0.013; **p < 0.01; ***p < 0.001.

Chronic neuroinflammation is driven by an overproduction of pro-inflammatory cytokines (Thakur et al., 2023). To assess the impact of chronic PEA treatment on the hippocampal inflammatory profile of Tg2576 mice, we performed a comprehensive protein microarray analysis targeting 40 distinct murine cytokines. As illustrated in Figure 5, significant alterations were observed in 5 cytokines. In particular, CXCL13, a chemokine responsible for attracting immune cells to the brain, displayed a markedly decreased expression after chronic PEA administration in Tg2576 mice (Tg Veh: 0.06 ± 0.02; Tg PEA 0.00 ± 0.00; *p* = 0.0008). Conversely, fractalkine (CXC3CL1), the chemokine and adhesion molecule with a known key dual role in neuroinflammation, showed a significant increase after PEA administration (Tg Veh: 0.14 ± 0.01; Tg PEA 0.37 ± 0.04; *p* < 0.005). G-CSF (Granulocyte-colony stimulating factor), a cytokine involved in granulocyte production, also showed a markedly decreased expression in Tg2576 mice after PEA treatment (Tg Veh: 0.04 ± 0.01; Tg PEA 0.02 ± 0.01; *p* = 0.003). Furthermore, interleukin-9 (IL-9), an immunoregulatory cytokine, exhibited a notable upregulation upon PEA treatment in Tg2576 mice (Tg Veh: 0.07 ± 0.03; Tg PEA 0.22 ± 0.08; *p* = 0.013). The expression of MCP-1 (Monocyte Chemoattractant Protein-1), a chemokine that attracts monocytes to sites of inflammation, displayed a significant decline upon chronic PEA administration (Tg Veh: 0.18 ± 0.06; Tg PEA 0.03 ± 0.01; *p* = 0.002).

### Chronic PEA administration reversed tyrosine nitrosylation in Tg2576 mice

Oxidative stress in the AD brain is evidenced by enhanced protein oxidation, notably characterized by the substantial rise in tyrosine residue nitration (Sultana et al., 2006; Alkam et al., 2007; Pacher et al., 2007). Here, we investigated the effect of chronic PEA administration on the levels of 3-nitrotyrosine (3-NT) in brain tissues.

**Figure 6:**
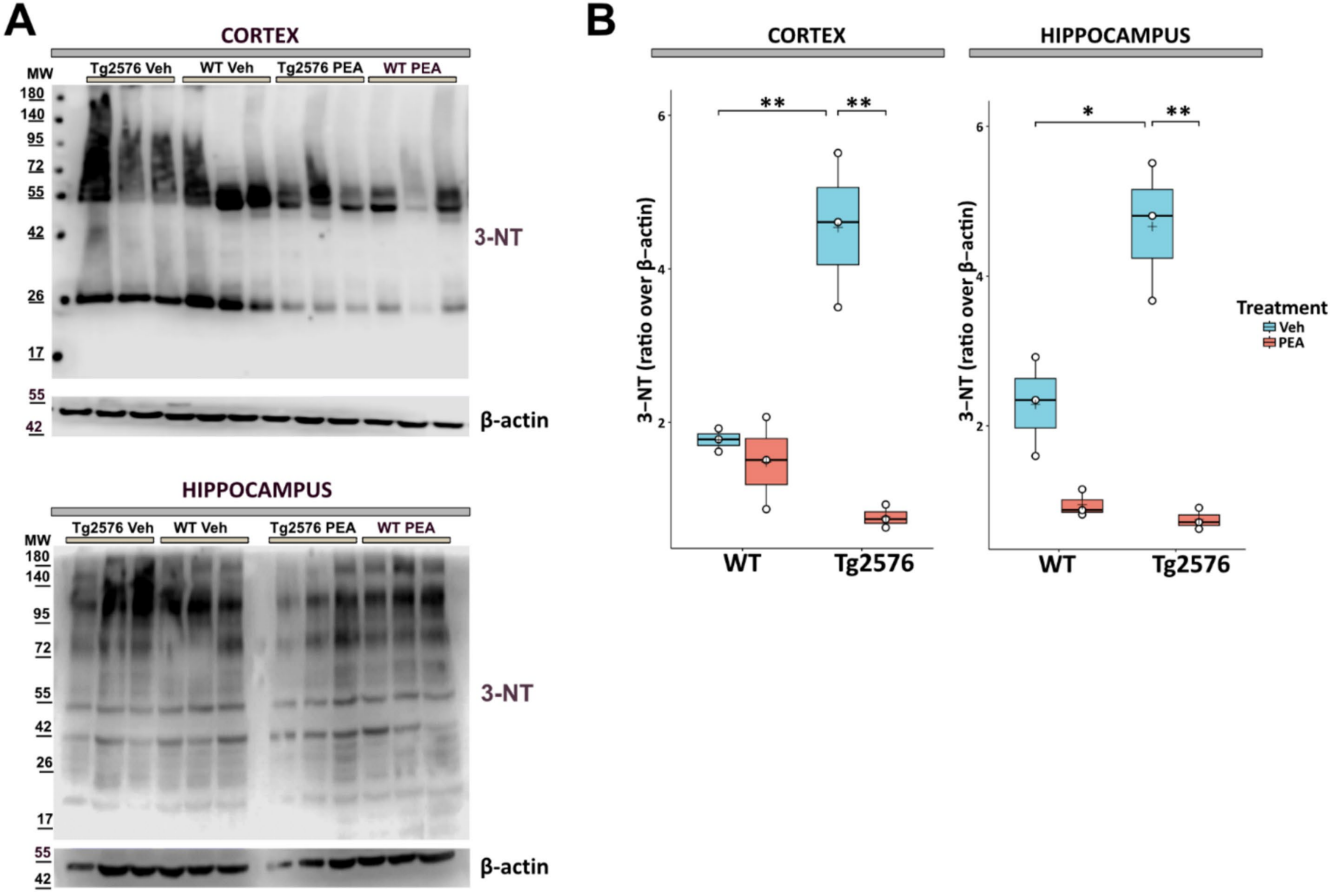
Expression of 3-nitrotyrosine (3-NT) in proteins isolated from the cortex and hippocampus of 12-month-old wild-type (WT) and Tg2576 mice upon chronic PEA administration. Western blot analysis of 3-NT in cortical and hippocampal extracts from WT and Tg2576 mice. β-actin was used as a loading control. **(A)** Representative immunoblots of 3-NT in the cortex (up) and hippocampus (down), MW: molecular weight (kDa); **(B)** Horizontal line and + within the rectangle (boxplot) represent the median and the mean, respectively, of densitometric analysis of lane values (white circles) of the amounts of 3-NT normalized to β-actin (*n* = 3 mice for each group). A two-way ANOVA was used to compare differences between treatment and genotype. Significance is shown as a *p* value, calculated using a post hoc Tukey’s test. *p = 0.02; **p < 0.01.

As shown in Figure 6, it was observed that 3-NT levels in Tg2576 mice were ~ 2 times higher than those of the corresponding WT mice across both assessed areas (cortex: Tg Veh: 1.48 ± 0.60; WT Veh: 1.77 ± 0.15; *p* = 0.0092; hippocampus: Tg Veh: 4.66 ± 0.92; WT Veh: 2.28 ± 0.66; *p* = 0.0226). Of note, prolonged PEA treatment resulted in a marked reduction of 3-NT in these regions of Tg2576 mice (cortex: Tg Veh: 4.54 ± 0.15; Tg PEA: 0.76 ± 0.15; *p* = 0.003; hippocampus: Tg Veh: 4.66 ± 0.92; Tg PEA: 0.74 ± 0.14; *p* = 0.001).

### Chronic PEA administration reduced the overexpression of inducible nitric oxide synthase in Tg2576 mice

**Figure 7:**
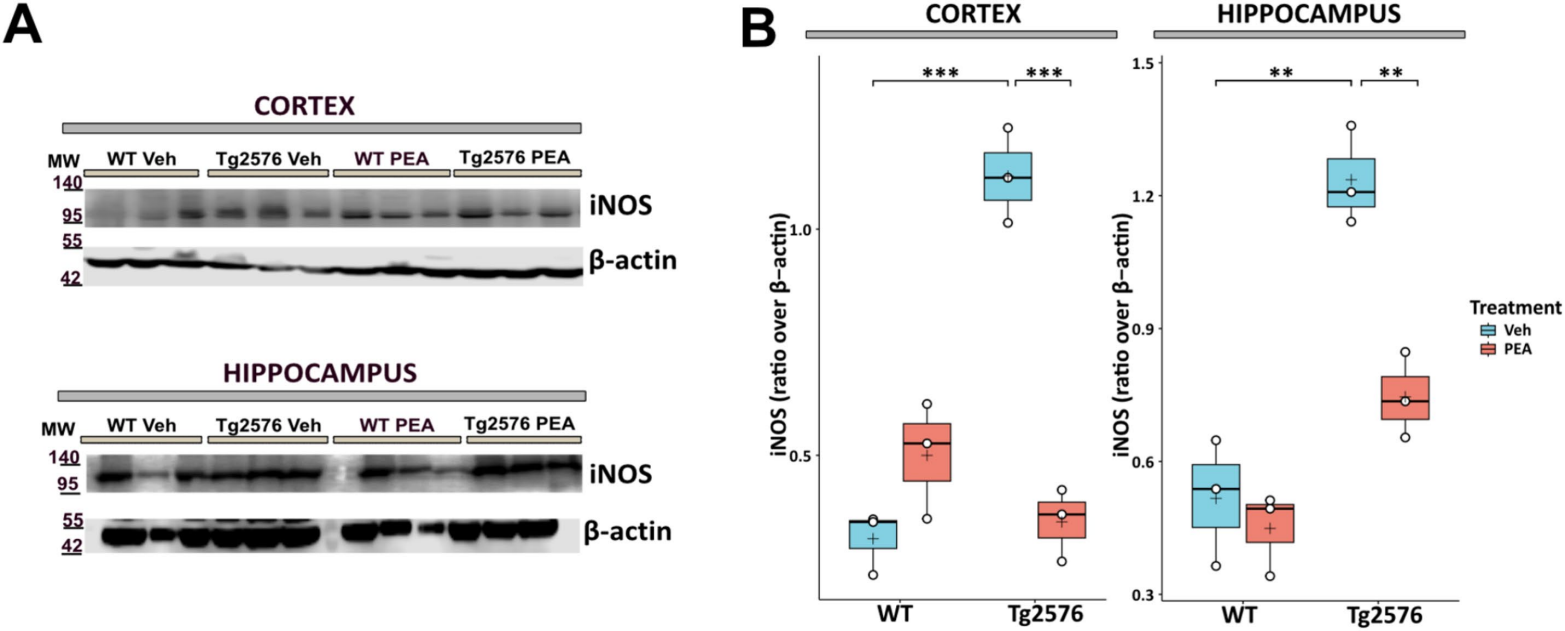
Expression of inducible nitric oxide synthase (iNOS) in cortex and hippocampus of 12-month-old wild-type (WT) and Tg2576 mice upon chronic PEA administration. Western blot analysis of iNOS in cortical and hippocampal extracts from WT and Tg2576 mice, β-actin was used as a loading control. **(A)** Representative immunoblots of iNOS in the cortex (up) and hippocampus (down), MW: molecular weight (kDa); blots were obtained from the same membrane used for Figure 3 after stripping and re-probing **(B)** Horizontal line and + within the rectangle (boxplot) represent the median and the mean, respectively, of the densitometric analysis values (white circles) of the amounts of iNOS normalized to β-actin (*n* = 3 mice for each group). A two-way ANOVA was used to compare differences between treatment and genotype. Significance is shown as a *p* value, calculated using a post hoc Tukey’s test. **p < 0.01; ***p < 0.001.

The upregulated activity of inducible nitric oxide synthase (iNOS) is known to play a role in the neuroinflammatory responses and neuronal damage that underline the progression of AD pathology (Asiimwe et al., 2016). Figure 7 illustrates that in Tg2576 mice, at the age of 12 months, there was a substantial increase in iNOS protein levels compared to WT mice (cortex: Tg Veh: 1.12 ± 0.11; WT Veh: 0.32 ± 0.07; *p* = 0.0004; hippocampus: Tg Veh: 1.24 ± 0.11; WT Veh: 0.52 ± 0.14; *p* = 0.0023). Interestingly, prolonged PEA treatment in Tg2576 mice resulted in a substantial reduction in iNOS protein levels within the cortex and hippocampus, as determined by Western blot analysis (cortex: Tg Veh: 1.12 ± 0.11; Tg PEA: 0.35 ± 0.08; *p* = 0.0005; hippocampus: Tg Veh: 1.24 ± 0.11; Tg PEA: 0.75 ± 0.10; *p* = 0.0045).

### Chronic PEA administration preserves synaptic function by downregulation of calcineurin expression in Tg2576 mice

**Figure 8:**
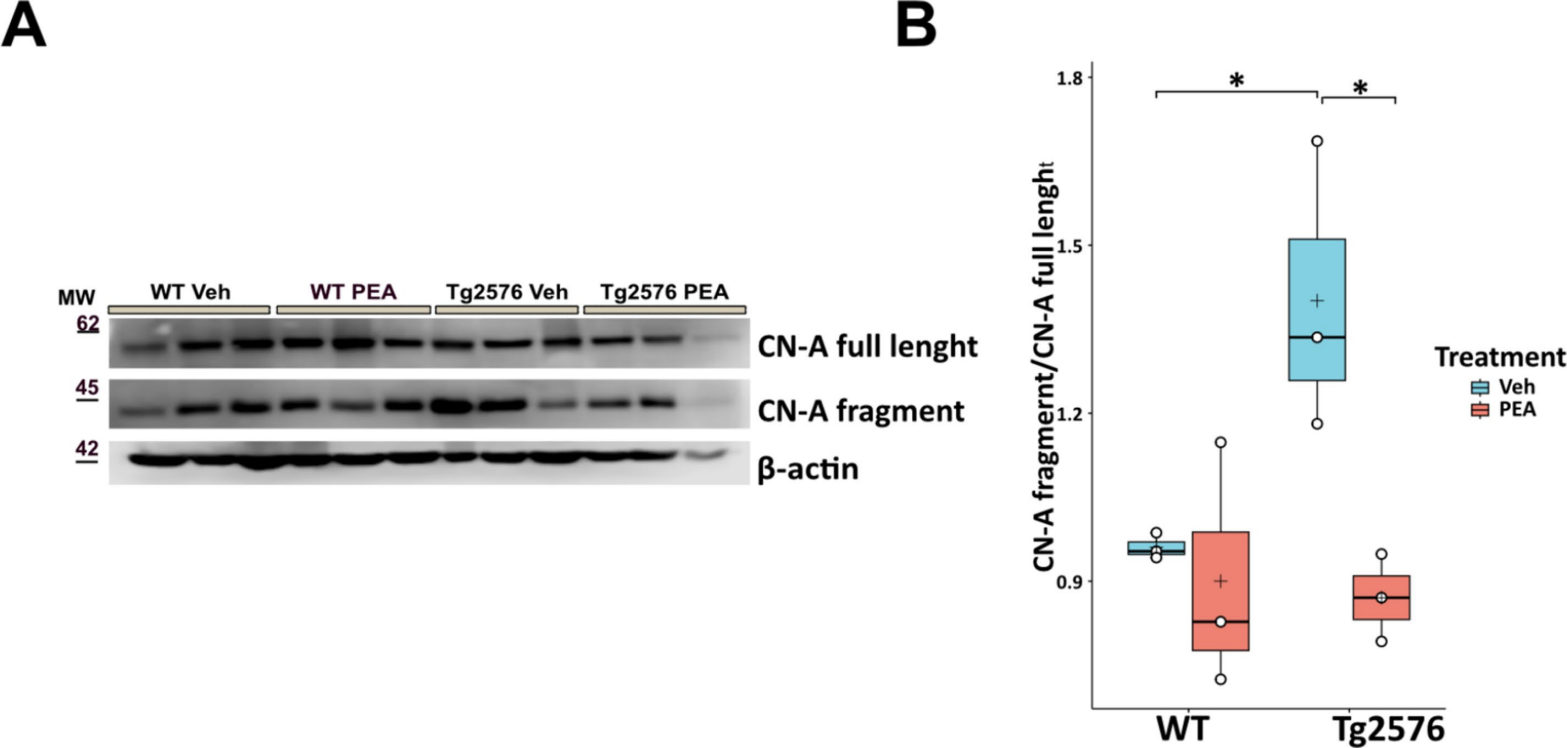
Expression of CN-A in the hippocampus of 12-month-old wild-type (WT) and Tg2576 mice upon chronic PEA administration. Western blot analysis of CN-A in hippocampal extracts from WT and Tg2576 mice, β-actin was used as a loading control. **(A)** Representative immunoblots of CN-A full length and its N-terminal fragment in Tg2576 hippocampus, MW: molecular weight (kDa); **(B)** Horizontal line and + within the rectangle (boxplot) represent the median and the mean, respectively, of the densitometric analysis values (white circles) of the amounts of CN-A full length and its N-terminal fragment normalized to β-actin (*n* = 3 mice for each group). A two-way ANOVA was used to compare differences between treatment and genotype. Significance is shown as a *p* value, calculated using a post hoc Tukey’s test. *p < 0.01.

Calcineurin (CN), a calcium/calmodulin-dependent serine/threonine phosphatase, plays a critical role in regulating dendritic spine density through its involvement in synaptic plasticity and structural remodeling (Reese and Taglialatela, 2011; Mackiewicz et al., 2023). Upon calcium influx through NMDA receptors and voltage-gated calcium channels, calcineurin activation leads to the dephosphorylation of cytoskeleton-reorganizing proteins, such as cofilin, destabilizing actin filaments within dendritic spines and promoting spine retraction. Additionally, calcineurin influences NFAT transcription factor activity, which regulates gene expression tied to synaptic plasticity and structural remodeling. High calcineurin activity/expression has been associated with reduced dendritic spine density, as it promotes the disassembly of actin filaments within the spines, thereby impacting both synaptic stability (Reese and Taglialatela, 2011; Mackiewicz et al., 2023). Thus, as shown in Figure 8, we analyzed the expression of calcineurin catalytic subunit A (CN-A) by Western blot analysis in hippocampal protein extracts of WT and Tg2576 mice, at the age of 12 months, after prolonged PEA or vehicle treatment. Tg2576 mice exhibited a significant elevation in CN-A protein levels at 12 months of age compared to WT controls (Tg Veh: 1.40 ± 0.26; WT Veh: 0.96 ± 0.02; *p* = 0.04). Remarkably, PEA treatment reduced the expression of calcineurin Tg2576 mice (Tg Veh: 1.40 ± 0.26; Tg PEA: 0.87 ± 0.08; *p* = 0.02; WT Veh: 0.96 ± 0.02; WT PEA: 0.90 ± 0.22).

### Chronic PEA administration reversed dendritic spine loss in Tg2576 mice

**Figure 9:**
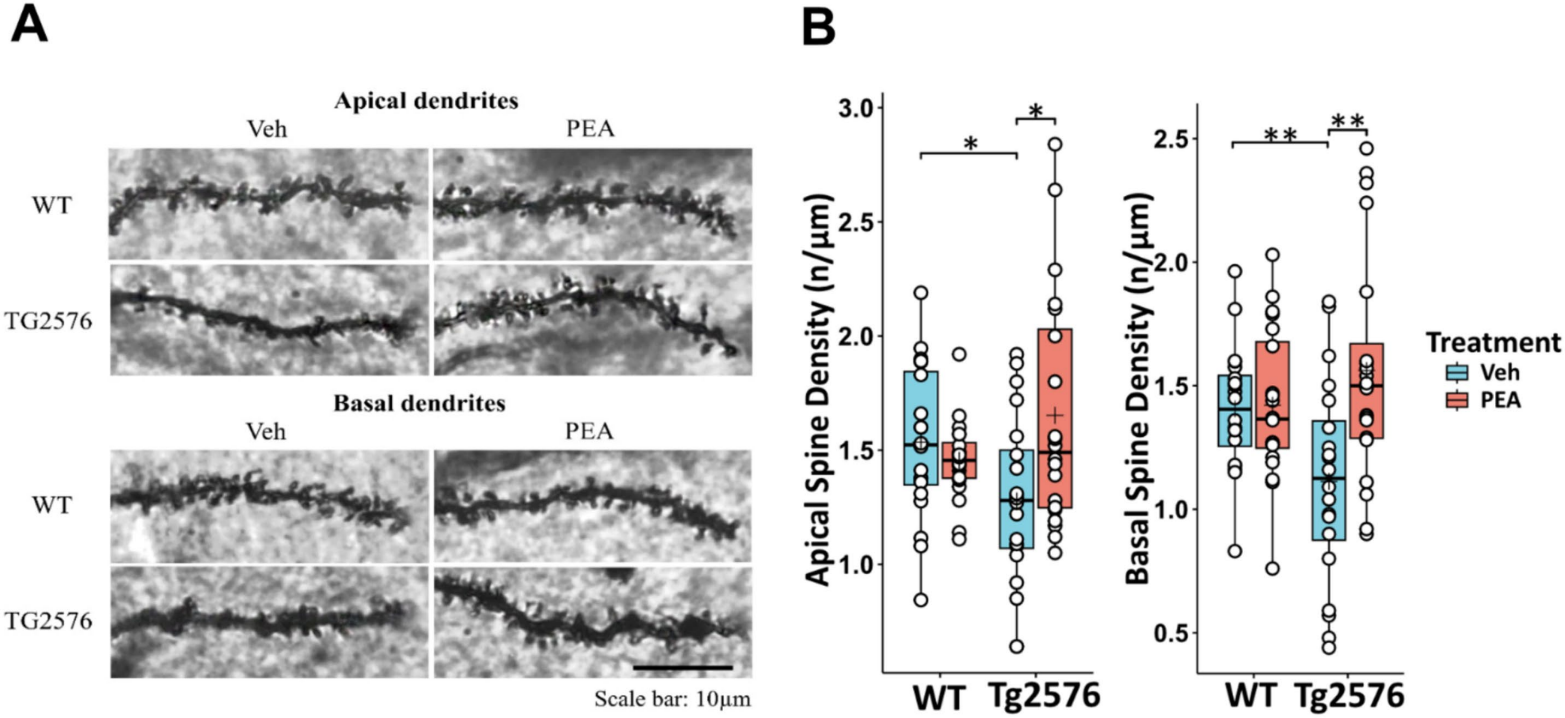
Evaluation of the hippocampal CA1 pyramidal neurons dendritic spine density of 12-month-old wild-type (WT) and Tg2576 mice upon chronic PEA administration. Spine quantification analysis (by Golgi-Cox staining) of apical and basal dendrites of hippocampal CA1 pyramidal neurons from WT and Tg2576 mice **(A)** Representative Golgi-stained images of the dendritic spines in hippocampus sections. **(B)** The horizontal line and + within the rectangle (boxplot) represent the median and the mean, respectively, of the measurement values (white circles) (*n* = 2 mice for each group, 10 pyramidal neuron measurements for mouse). A two-way ANOVA was used to compare differences between treatment and genotype. Significance is shown as a *p* value, calculated using a post hoc Tukey’s test. *p < 0.05; **p < 0.01.

AD is characterized by the loss of neurons and synapses, particularly in the hippocampus (Mangalmurti and Lukens, 2022), and a key feature of neurodegeneration in AD is the disruption of dendritic spine density (Dorostkar et al., 2015; Mangalmurti and Lukens, 2022). To evaluate the potential neuroprotective effects of chronic PEA administration in Tg2576 mice, we quantified dendritic spine density by using Golgi-Cox staining. Consistently with the observed pathological changes, Tg2576 mice displayed a significant reduction in spine density of hippocampal CA1 pyramidal neurons, affecting both apical and basal dendrites compared to their WT littermates (apical: WT Veh: 1.53 ± 0.34 n/μm; Tg Veh: 1.28 ± 0.38 n/μm; *p* = 0.027; basal: WT Veh: 1.41 ± 0.25 n/μm; Tg Veh: 1.11 ± 0.41 n/μm; *p =* 0.01) (Figure 9). Remarkably, chronic PEA treatment led to a substantial restoration of spine density in CA1 pyramidal neurons, bringing spine numbers close to those observed in WT mice (apical: Tg Veh: 1.28 ± 0.38 n/μm; Tg PEA: 1.65 ± 0.52 n/μm; *p =* 0.012; basal: Tg Veh: 1.11 ± 0.41 n/μm; Tg PEA: 1.56 ± 0.48 n/μm; *p* = 0.026).

### Chronic PEA administration ameliorated cognitive performance in Tg2576 mice

**Figure 10:**
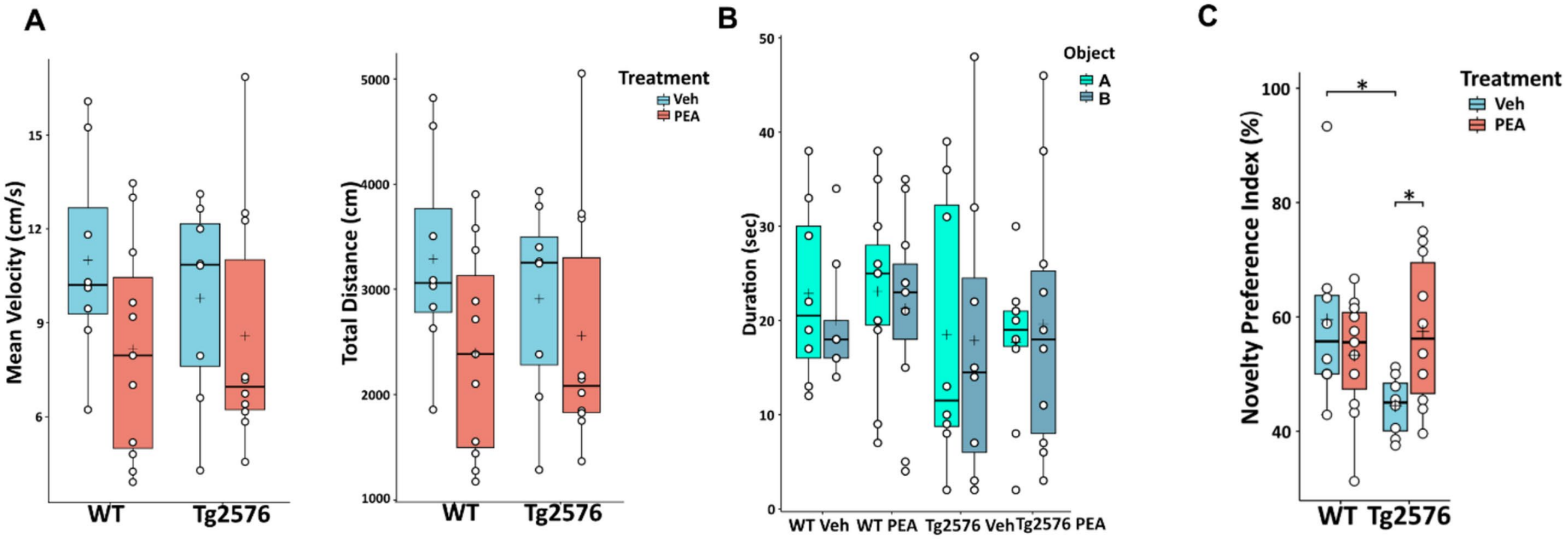
Novel Object Recognition (NOR) test of 12-month-old WT and Tg2576 mice upon chronic PEA administration. The horizontal line and + within the rectangle (boxplot) represent the median and the mean, respectively, of the values (white circles) which represent individual mice (*n* = 8 for vehicle groups; *n* = 11 for WT PEA; *n* = 10 for Tg PEA). **(A)** Mean velocity (cm/s) and total distance traveled by the mice in the habituation phase, **(B)** Duration of exploration of the two identical objects (object **A**, object **B**) in the training phase, **(C)** Novelty Preference Index scored in the test trial. A two-way ANOVA was used to compare differences between treatment and genotype. Significance is shown as a *p* value, calculated using a post hoc Tukey’s test. *p < 0.05.

All animals whose brains were utilized for biochemical and histological analyses were previously subjected to behavioral testing using the Novel Object Recognition (NOR) test. In the habituation phase, no differences between groups were found in mean velocity and total distance traveled (Figure 10A). Moreover, in the training phase, no differences between groups were found in the exploration of the two identical objects, allowing the exclusion of possible spatial or object preference bias (Figure 10B). In the test trial shown in Figure 10C, in comparison to WT mice, Tg2576 mice exhibited impaired cognitive performance, which was characterized by a significant reduced ability to discriminate between novel and familiar objects (Novelty Preference Index: WT Veh: 59.50 ± 15.55; Tg Veh: 44.46 ± 5.17; *p* = 0.0212).

Remarkably, chronic PEA administration rescued cognitive impairment in Tg2576 mice. Indeed, PEA treatment restored the recognition memory performance of Tg2576 mice, which displayed values similar to those of both WT groups (Tg Veh: 44.46 ± 5.17; Tg PEA: 57.47 ± 12.29; *p* = 0.0171; WT Veh: 59.50 ± 15.55; WT PEA: 53.30 ± 10.34).

## Discussion

In this study, we explored the potential therapeutic effects of chronic PEA administration in the Tg2576 mice. Our findings indicate that chronic PEA treatment (i) exerts an anti-amyloidogenic action by inducing ADAM9 expression, (ii) reduces astrogliosis, (iii) influences microglial phenotype and modulates neuroinflammation by downregulating critical chemokines involved in immune cell recruitment and upregulating anti-inflammatory chemokines, (iv) mitigates oxidative stress, (v) preserves synaptic function by reversing the loss of both basal and apical dendritic spine density in CA1 pyramidal neurons along with calcineurin downregulation, and (vi) alleviates memory deficits observed in Tg2576 mice.

Previous studies demonstrated that ultra-micronized PEA formulations enhance the rate of dissolution and reduce absorption variability reported for the orally administered compound (Cocito et al., 2014; Impellizzeri et al., 2014). In addition, pre-clinical studies demonstrated that subcutaneously implanted PEA pellets are significantly more effective than oral PEA administration in mouse models (Scuderi et al., 2018; Bellanti et al., 2022). Such an enhanced efficacy is likely due to several factors, e.g., the ability of PEA particles to bypass liver metabolism. Our data confirm that chronic PEA treatment *via* subcutaneous pellets effectively increases circulating and brain PEA levels in both WT and Tg2576 mice, with the brain of the latter showing more pronounced effects.

We demonstrate that chronic administration of PEA induces the hippocampal expression of ADAM9 in Tg2576 mice, which is known to promote the non-amyloidogenic processing of amyloid precursor protein (APP) through the activation of α-secretase activity (Asai et al., 2003; Chou et al., 2020). This mechanism reduces the formation of amyloid-beta (Aβ) peptides, suggesting that PEA administration could facilitate the non-amyloidogenic cleavage of APP, thereby protecting neurons from amyloid-induced toxicity.

In addition, our findings support that chronic PEA administration mitigates amyloid-induced astrogliosis in the cortex and hippocampus of Tg2576 mice consistently with previous *in vitro* and *in vivo* studies showing the decrease of GFAP protein expression after PEA treatment (Scuderi et al., 2011, 2014). In the present study, Tg2576 mice exhibited a reactive hyper-ramified microglia, characterized by a higher number of intersections, nodes, endpoints and longer processes (Hinwood et al., 2012, 2013; Smith et al., 2019; D’Aloia et al., 2021; Li et al., 2023), compared to WT controls.

This is consistent with several studies demonstrating that resting-state, ramified microglia, when stimulated by various insults, such as beta-amyloid, shift into an intermediate ‘reactive’ phenotype. In this state, referred to ‘hyper-ramified’ state, microglia cells are characterized by an increase in the number and volume of processes, and are capable of secreting cytokines and chemokines, including pro-inflammatory mediators (Walker et al., 2014; Holloway et al., 2019). Notably, chronic PEA administration attenuated microglia reactivity in Tg2576 mice, thus promoting WT-like homeostatic microglia. Our findings suggest that the chronic PEA treatment exerts its beneficial effects by restoring a quiescent state of microglial cells in the context of reduced neuroinflammation and oxidative stress. The morphological changes in microglial cells observed here are in line with previous *in vitro* findings, showing that PEA treatment following inflammatory stimulation with LPS exerts neuroprotective and anti-inflammatory effects through the modulation of microglia reactive phenotype (D’Aloia et al., 2021). Interestingly, hyper-ramifications of glial cells have been recently demonstrated to be associated with dendritic spine loss in the neurons of hippocampus and medial prefrontal cortex, in a mouse model of post-traumatic stress disorder (Smith et al., 2019). Aβ1–42 oligomers promote neuroinflammation and neuronal death in AD brain by eliciting the release of proinflammatory cytokines (IL-1β and TNF-α) from microglia. By interfering with the synthesis of anti-inflammatory cytokines (Leng and Edison, 2021; Sorrentino et al., 2021), TNF-α inhibits microglia phagocytosis of Aβ and stimulates γ-secretase activity, thus facilitating Aβ accumulation and microglia-mediated neuroinflammation (Leng and Edison, 2021; Sorrentino et al., 2021). Therefore, we investigated the expression of key pro- and anti-inflammatory cytokines in the hippocampus of Tg2576 mice upon chronic PEA treatment. For the first time, we showed that chronic PEA treatment significantly reduces the expression of CXCL13, a selective chemoattractant for B1 and B2 cells (two sub-classes of B cell lymphocytes involved in the humoral immune response), and elicits its effects by interacting with the CXCR5 receptor that is expressed by microglia and T and B lymphocytes. In a recent study, elevated levels of CXCL13 were identified in the cerebrospinal fluid and brain tissue of authentic AD patients (Bivona et al., 2023). Such an increase correlated with worse cognitive function and neurodegeneration, suggesting that CXCL13 may play a significant role in the development of AD. In addition, in the 3xTg AD mice model microglial re-population following depletion led to increased CXCL13 expression, with differential changes in tau phosphorylation and amyloid pathology (Karaahmet et al., 2022).

Similarly, the observed downregulation of MCP-1, a chemokine responsible for monocyte recruitment to sites of inflammation, and the decreased G-CSF expression in Tg2576 mice following PEA administration suggest that PEA may dampen inflammatory cell infiltration and have a mitigating effect on granulocyte production. Collectively, these findings indicate that chronic PEA treatment may exert an overall anti-inflammatory effect within the hippocampus of Tg2576 mice. Interestingly, our data revealed for the first time, increased fractalkine (CXC3CL1) and IL-9 expression after chronic PEA administration. CX3CL1/CX3CR1 signaling pathway modulates microglial responses to stimuli, including Aβ deposition, and plays a critical role in regulating microglial phenotype (Karaahmet et al., 2022; Guadalupi et al., 2024). Furthermore, fractalkine signaling influences synaptic plasticity and promotes adult hippocampal neurogenesis, both of which are crucial in learning and memory processes that are impaired in AD (Finneran and Nash, 2019). However, the impact of fractalkine on neurodegeneration still appears controversial, disruption of its signaling being beneficial in some disease states (e.g., amyloid pathology and stroke) and yet detrimental in other neurodegenerative diseases (e.g., Parkinson’s disease, amyotrophic lateral sclerosis, and tauopathies) (Finneran and Nash, 2019). The involvement of IL-9 in AD is still unexplored, even though a recent study on an experimental autoimmune encephalomyelitis (EAE) model of multiple sclerosis highlighted IL-9 as a critical neuroprotective molecule, capable of interfering with inflammatory synaptopathy (Guadalupi et al., 2024). Specifically, in the EAE model, IL-9 modulated microglial inflammatory activity by both enhancing the expression of the trigger receptor expressed on myeloid cells-2 and reducing neuronal TNF-α release and signaling thereof, thus blocking its synaptotoxic effects.

Different studies reported that oxidative and nitrosative damage may be important in the pathogenesis of AD (Sultana et al., 2006; Alkam et al., 2007; Pacher et al., 2007). In the present investigation, we showed that chronic PEA administration leads to a marked reduction of protein nitrosylation in the cortex and hippocampus of Tg2576 mice. These findings align with previous data demonstrating that daily PEA administration for 1–2 weeks in mice injected intracerebroventricularly with amyloid-β 25–35 reduced lipid peroxidation and protein nitrosylation, in both WT and PPAR-α^−/−^ mice (D’Agostino et al., 2012). Oxidative and nitrosative stress are widely recognized as critical factors in the pathogenesis and progression of AD (Butterfield and Boyd-Kimball, 2020), with the mitochondria as their major source. This seems relevant because in young and pre-plaque stage (i.e., 3-month-old) Tg2576 mice an altered activity of mitochondria function was described that culminated in cytochrome c release and active caspase-3 accumulation in hippocampal postsynaptic compartments (D’Amelio et al., 2011).

Likewise, our findings reinforce the evidence already demonstrated by others that chronic PEA administration in Tg2576 mice can downregulate iNOS protein expression (Scuderi et al., 2011, 2014), which is directly involved in oxidative stress and mitochondrial function. A previous study, showed that caspase-3 activates calcineurin which, in turn, triggers the dephosphorylation and the removal of the GluR1 subunit of AMPA-type receptor, leading to altered postsynaptic density and dendritic spine loss (D’Amelio et al., 2011). The inhibition of caspase-3 or calcineurin, through Z-DEVD-FMK (D’Amelio et al., 2011) or FK506 (Cavallucci et al., 2013) respectively, completely rescued the dendritic spine loss and cognitive deterioration. Of note, in an amyloid-induced neurotoxicity mouse model, a five-day treatment with PEA significantly decreased caspase-3 protein expression (D’Agostino et al., 2012). In our study, we interestingly demonstrate for the first time that the reduction of oxidative stress following PEA chronic treatment is also accompanied by a decrease in calcineurin expression in the hippocampus of Tg2576 mice. These observations are quite overlapping to the morphological arrangement we have found in the hippocampus of Tg2576 mice, where the microglial reactive phenotype was indeed present together with loss of apical and basal dendritic spines of CA1 pyramidal neurons. Notably, in Tg2576 mice chronic PEA administration rescued the WT-like microglial morphology and protected CA1 pyramidal neurons from the loss of apical and basal dendritic spines. Experimental evidence supports the concept that the immune response modulates synaptogenesis and/or restores neuronal connectivity upon an acute or chronic damage (Dantzer, 2018). The modulatory role of the immune system is strictly linked to immune mediators, such as cytokines, which modulate synaptic transmission and alter the morphology of dendritic spines during the inflammatory process following brain injury. As noted above, PEA remarkably changes the immunophenotype of Tg2576, and here we proved that it is also able to modify hippocampal plasticity by acting on dendritic spine arborization.

It can be stressed that the effects of PEA on dendritic spine density, that are described here for the first time, as well as those on the morphology of glial cells could be mediated by an increased expression of peroxisome proliferator-activated receptor-α (PPAR-α) and/or by its translocation from the cytoplasm to the nucleus of microglia cells. PPAR-α in turn decreases: (i) production of nitric oxide (NO), (ii) influx of immune cells, and (iii) expression of pro-inflammatory proteins like iNOS and TNF-α. Ultimately, PEA may exert its protective function, maintain dendritic spine density, and promote polarization of microglia into the anti-inflammatory phenotype, by inhibiting the generation of inflammatory cytokines (IL-1β and TNF-α included) through the PPAR-α/NF-kB pathway (Rankin and Fowler, 2020).

Accordingly, several studies have shown that PPAR-α is highly expressed in the hippocampus and is engaged in: (i) the regulation of cyclic AMP response element-binding (CREB) protein, (ii) the modulation of synaptic plasticity, and (iii) learning and memory functions (Roy et al., 2013, 2015; Roy and Pahan, 2015; Rankin and Fowler, 2020).

Moreover, our data support that PEA can reinstate synaptic plasticity and function in hippocampal neurons, providing the morphological basis of its neuroprotective effects and ability to recover cognitive performance. In fact, the hippocampus (and more specifically its CA1 subarea) is a pivotal brain region intricately involved in learning and memory processes, and has been identified as a significant contributor to object recognition memory in rodents (Stackman et al., 2016; Ásgeirsdóttir et al., 2020). Consistently, here chronic PEA administration protected Tg2576 mice from cognitive impairment in the NOR test, confirming previous studies with different animal models, routes, durations and methods of PEA administration (D’Agostino et al., 2012; Scuderi et al., 2018).

## Conclusion

This study investigated the therapeutic potential of chronic palmitoylethanolamide (PEA) administration in the Tg2576 mouse model of Alzheimer’s Disease (AD). Our findings demonstrate that chronic PEA administration via subcutaneous pellets exerts multiple neuroprotective effects. Specifically, PEA administration may facilitate the non-amyloidogenic cleavage of amyloid precursor protein (APP) and attenuate neuroinflammation by suppressing microglia and astrocyte activation, thereby reducing pro-inflammatory cytokine release within Tg2576 hippocampus. Furthermore, PEA may preserve synaptic integrity by mitigating oxidative and nitrosative stress, potentially through the downregulation of inducible nitric oxide synthase (iNOS). By potentially inhibiting calcineurin, PEA may prevent GluR1 subunit dephosphorylation and AMPA receptor removal, thus attenuating dendritic spine loss and synaptic dysfunction, and counteracting cognitive decline. Taken together, these findings support the therapeutic potential of chronic PEA administration for AD patients, encouraging further exploration of PEA mechanisms of action. However, elucidating a singular, definitive mechanism remains challenging due to PEA’s pleiotropic effects and interactions with diverse cellular targets and signaling pathways in this neurodegenerative disorder.

## Data Availability

The raw data supporting the conclusions of this article will be made available by the authors, without undue reservation.
